# Thinking BIG rheumatology: how to make functional genomics data work for you

**DOI:** 10.1186/s13075-017-1504-9

**Published:** 2018-02-12

**Authors:** Deborah R. Winter

**Affiliations:** Department of Medicine, Division of Rheumatology, Northwestern University Feinberg School of Medicine, Chicago, IL 60611 USA

**Keywords:** Functional genomics, Big data, High-throughput sequencing assays, Gene expression, Epigenomics, Chromatin

## Abstract

High-throughput sequencing assays have become an increasingly common part of biological research across multiple fields. Even as the resulting sequences pile up in public databases, it is not always obvious how to make use of these data sets. Functional genomics offers approaches to integrate these "big" data into our understanding of rheumatic diseases. This review aims to provide a primer on thinking about big data from functional genomics in the context of rheumatology, using examples from the field’s literature as well as the author’s own work to illustrate the execution of functional genomics research. Study design is crucial to ensure the right samples are used to address the question of interest. In addition, sequencing assays produce a variety of data types, from gene expression to 3D chromatin structure and single-cell technologies, that can be integrated into a model of the underlying gene regulatory networks. The best approach for this analysis uses the scientific process: bioinformatic methods should be used in an iterative, hypothesis-driven manner to uncover the disease mechanism. Finally, the future of functional genomics will see big data fully integrated into rheumatology, leading to computationally trained researchers and interactive databases. The goal of this review is not to provide a manual, but to enhance the familiarity of readers with functional genomic approaches and provide a better sense of the challenges and possibilities.

## Background

The medical sciences are abuzz with big data. Across virtually every field and discipline there are projects to collect data from “omics” assays. The resulting data sets are characterized as “big data” and may involve thousands to billions of measurements for a handful to thousands of individual samples. In the field of functional genomics, the traditional omics assay makes use of next-generation sequencing technology. Such assays tend to relate to genomics, transcriptomics, and epigenomics although these data sets may be linked to everything from metabolomics and proteomics to microbiomics and clinical “omics”. These high-throughput assays generate libraries of DNA fragments that are “read” by the sequencer. These sequence reads must be computationally linked to generate a de novo genome (usually in the case where the source genome is unknown) or aligned to the reference genome. In the case of patient data, this means aligning millions or billions of reads—depending on the desired coverage—to the approximately 3 billion base-pair human genome. At later stages, these data may be simplified into summary files listing only the genes or regions of interest: however, the final list is still likely to contain thousands of entries. Although the simplest experiment may only have two conditions each with a few replicates, it is rapidly becoming common for studies to include several populations under changing conditions or across time. Moreover, studies involving human patients may wish to include large cohorts to address individual variation. Finally, with the expanding popularity and ease of single-cell technologies, each individual cell may represent its own sample and may number in the thousands and counting.

Several consortiums exist that collect these types of big data in a systematic way and make it publicly available. The ENCyclopedia of DNA Elements (ENCODE) is an example of a consortium that seeks to build a comprehensive list of functional elements in the human genome [1]. The ENCODE pilot began with 1% of the genome across a small defined set of common human cell lines [2], but it rapidly expanded to include a variety of cell types and then spun off into the Epigenomics Roadmap to catalog human variation by collecting tissue samples from many individuals [3]. Together, ENCODE and Roadmap represent data from gene expression, protein binding, chromatin state, and other assays and are accompanied by a host of quality standards, computational tools, and annotations resulting from the processing and analysis [4]. Another consortium, Genotype-Tissue Expression project (GTex), focuses specifically on the interactions between genotype and gene expression in vivo by collecting multiple tissues from human donors [5]. In contrast, the NIH Library of Integrated Network-based Cellular Signatures (LINCS) is cataloging the generalized response profiles by measuring changes in gene expression in multiple cell types exposed to a variety of induced perturbations [6]. For immunology specifically, the Immunological Genome Project (ImmGen) [7] and the European Blueprint Epigenome [8] provide databases for gene expression and epigenomics, respectively. These examples are valuable resources of quality-controlled data that may obviate the requirement to repeat assays in individual laboratories. However, it is one thing to catalog these data and another to use them to test a hypothesis. This is the difference between descriptive genomics and functional genomics.

Functional genomics makes use of high-throughput sequencing data to describe how genes contribute to the identity, function, and activity of cells. It is often used interchangeably with the term “systems biology” because both tend to involve modeling of multiple interacting components and use a variety of techniques from different fields to study the resulting networks. In functional genomics, the collection of interactions can be described as the Gene Regulatory Network (GRN) of the cell. Gene expression as reflected by RNA transcription is the typical output of GRNs while the interactors are cis-regulatory elements within the noncoding genome and the transcription factors (TFs) that bind them. Because the transcription of RNA is not perfectly correlated with protein translation, it may not directly reflect the cell’s phenotype. However, the cell’s GRN is critical to understanding the underlying programming of the cell regardless of the exact impact on function. High-throughput sequencing assays provide the raw data with which to model these GRNs. The goal of functional genomics is to inform how cells evolved to fulfill their roles and the pathways to which they devote their energy.

The aim of this review to provide a primer on big data in functional genomics in the context of rheumatology. Although other articles cover similar topics, this review is meant to be accessible to researchers whose focus is rheumatic disease by highlighting field-specific issues and using examples from the literature. It is not intended to present a step-by-step manual, but rather to draw attention to key features of big data studies and provide information on the critical techniques. The first section discusses several options that should be considered when designing a functional genomics study to optimize the relevance to the question. Next follows a description of functional genomic data types and the assays commonly used to generate these data. In these studies, how the data are analyzed is as important as how they are collected. Thus, the third section gives an overview of the key approaches involved in bioinformatic analysis. Finally, the author shares her vision for the future of big data in rheumatology. For a precise description of the methods mentioned, please refer to the specific references throughout the text. For the most part, the goal is to present studies in rheumatology as examples, but other functional genomics research is used in order to cover a wider variety of approaches. Due to space constraints, the review does not cover some topics in functional genomics in detail—including noncoding RNA, splicing, and other post-transcriptional regulation. While not all relevant studies could be mentioned, the goal is to convey the big potential associated with these approaches and increase awareness of the possibilities and considerations involved.

## Main text

### *Big* study design

The key to successful research in functional genomics is designing the study to effectively address the question of interest. This is true even when the data are downloaded from a public database. However, in some cases it might be necessary to generate new data in order to have access to the most relevant data. In the world of functional genomics, study design boils down to what populations of cells are chosen as samples for the assay. Here, the major choices involved are discussed.

#### In vitro vs in vivo

This review refers to cell populations assayed directly after isolation from either the patient or the model organism as in vivo samples. Alternatively, cells may be first cultured or differentiated in vitro. The main advantage of in vitro populations is the ease in obtaining enough cells for the desired assay. For example, bone-marrow-derived macrophages (BMDMs) are commonly used to test hypotheses about immune response [9, 10]. However, recent work has shown systematically that BMDMs and the related dendritic cells (DCs) that are differentiated by culturing bone marrow cells with granulocyte–macrophage colony-stimulating factor (GMCSF) do not fully resemble any in vivo population [11]. Moreover, even the GRN of mature cells are influenced by their local environment: thus, cells such as macrophages, when removed from the culture, tend to lose their distinct regulatory landscape [12, 13]. Similarly, Gardner et al. [14] showed that gene expression profiles obtained using explanted fibroblasts from patients and controls are a poor substitute for skin biopsies. There may be cases where assays on in vitro cells may be sufficient, or even preferable, for the question of interest. Using cultured cells may help distinguish between cell-autonomous and environmental effects. Also, in vitro cells are useful for proof-of-principle studies and to establish gene editing.

#### Mouse model vs patient sample

In rheumatology, mouse models are commonly used to approximate human diseases, such as systemic lupus erythematosus (SLE), systemic sclerosis (SSc), and rheumatoid arthritis (RA) [15–17]. Researchers have studied gene expression profiles of both genetic models, such as NZB/W mice that are SLE prone [18, 19], and chemical models, such as bleomycin-induced pulmonary fibrosis [20, 21]. Mouse models have the obvious advantage of being easier to manipulate and genetically identical mice minimize the individual variation. In addition, cells from any tissue or organ may be obtained for study at any stage of disease. On the other hand, it is often difficult to obtain human tissues, other than blood, at multiple time points. For example, skin biopsies have been used to study gene expression in SSc patients and controls [22], although the small sample size may limit the ability to study individual cell populations without first culturing the cells (e.g., fibroblasts [14]). Alternatively, researchers can take advantage of transplants to access donor and explanted tissue [23]; however, these samples typically represent end-stage disease. Several studies have shown high homology between both the definition of cell populations [24, 25] and their key regulatory factors [26] across mice, human, and other primates. Furthermore, kidney mononuclear cells from mouse models of lupus demonstrated significant overlap of GRNs and activated genes with human lupus nephritis samples [27]. The main shortcoming of mouse models arises for studies interested in the causative signals behind rheumatic disease; there is often a lack of evidence that the conditions by which symptoms were initiated in mice will reflect the real etiology of disease. For a given study, one must be clear on whether the intention is to study the cause or behavior of cells in disease.

#### Whole tissue vs cell-type specific

For the purposes of bulk assays in functional genomics, it is generally preferable to aim for homogeneous cell populations as opposed to whole tissue or mixed populations. Notably, single-cell technologies are the exception to this rule, as will be explained in the following. In bulk assays of hundreds to millions of cells, the resulting data represent an average across all cells and it is difficult to parse out the effect of specific cell types. Without sophisticated algorithms, it is impossible to distinguish between changes in expression within a single cell type and changes to the proportion of that cell type within the population. Using a network approach in a meta-analysis of other SSc expression studies, Mahoney et al. [28] implicated the interferon pathway, M2 macrophage polarization, adaptive immunity, and cell proliferation. However, while enriched pathways may be found in tissue samples, one cannot be sure that any group of genes is expressed in the same cell type. Moreover, it is conceivable that different cell types may regulate genes in opposite directions that then cancel each other out. Similarly, in our studies of RA, we have observed that changes in some less highly expressed genes within a single population, such as macrophages, may be drowned out by the remaining population in whole joint synovium (Mandelin et al, accepted). Thus, it is not feasible to model a coherent GRN of the interactions between genes and regulatory elements. Even with deconvolution, one must define a characteristic set of genes to attribute to each discoverable cell type [29, 30]. This approach may reveal the proportion of different cell types within the sample, but cannot then define the changes in the gene expression within these cell types without begging the question, since this data was used in the model's assumptions. Thus, in order to model the GRN of a cell type, each study should aim to assay the purest populations of cells possible.

#### Control, reference, and outgroup

A key aspect of study design in functional genomics is to choose one or more auxiliary samples that will underscore the feature of interest in the test sample. In the most common and straightforward cases for studying rheumatic disease, the test sample would be derived from patients while the control sample would come from healthy volunteers. This approach is seen in a study of lung tissue samples from patients with SSc and healthy controls [31]. Similarly, the mouse equivalent would compare the mouse model with mice given sham treatment of the same age and genetic background as the control. In the case of bleomycin treatment, the control mice would be intubated with PBS as in traditional experiments measuring changes in surface marker expression [21]. In contrast, the reference is defined as a sample that provides necessary context for an experiment. This may be the sample from “Day 0” in a time course of serum-transfer induced arthritis (STIA) vs control [25]; the population of monocytes that is thought to be the precursor of macrophages in SSc-associated skin fibrosis vs healthy skin [32]; or skin biopsies from controls in a study of response to mycophenolate in SSc patients [33]. These populations are assayed in addition to the typical control as they may be necessary to distinguish between regulatory factors that are involved in the main comparison vs other confounding variables. In another instance, skin samples from the unaffected back of SSc patients were used as a reference to define “intrinsic” genes that exhibited consistent expression with their affected forearm before comparison across patients [22]. Finally, in some experiments it may be helpful to include a population of cells as an outgroup. The purpose of an outgroup is to provide perspective about the relationship between the main population of a comparison. For example, in comparing tissue-resident macrophages, we included neutrophils as an outgroup to demonstrate the difference between macrophages compared to that between cell types that shared a common myeloid lineage [13]. As experiments become more complicated and involve multidimensional comparisons, the distinction between controls, references, and outgroups may be blurred. How these experimental tools are used should be evaluated on a case-by-case basis.

#### Replicates and batch effect

Once the identities of the comparison samples are chosen, one should consider including replicates whenever possible. The statistical power of high-throughput sequencing assays is relatively insensitive to the replicate number because they involve millions of measurements for thousands of genes and regulatory elements. Any single gene may fall below the cutoff value for the false discovery rate (FDR), but, globally, the set of differential genes represents a substantial effect size [34, 35]. Nonetheless, replicates are crucial to account for technical variation, experimental error, signal-to-noise ratio, and batch effect. Biological replicates generally refer to distinct samples either taken subsequently from the same patient or from genetically identical mice undergoing the same treatment. These can be used to account for variability in the experimental conditions and can help to control for artifacts in the data. On the other hand, a technical replicate occurs when a single sample is divided for the purposes of the assay. A technical replicate can be useful, especially when it is difficult or cost-prohibitive to obtain biological replicates of human samples, to estimate the amount of noise or to help optimize library protocols during development [36]. Another use of replicates is to provide robust results: the activity of a tissue-specific macrophage cis-regulatory element may be confirmed by enrichment in two replicates [13] and the identification of immune cell subsets in human blood can be validated across patients [37]. The other benefit of multiple replicates is to control for batch effect. The batch effect describes technical variation that arises between groups of samples that are prepared at different times. While this may sometimes be unavoidable based on the availability of samples for the throughput of the experiment, replicates can help to mediate the impact on the data. For both bulk and single-cell genomic assays, replicates should be spread out across groups so that batch effects are not confounded with the differences between the samples being compared [38, 39]. For example, in a single-cell study of microglia development, we replicated the single-cell population for each time point so that we could distinguish time-dependent differences from the batch effect [40]. Alternatively, long-term studies, such as longitudinal patient studies, may choose to use a reference sample (some are commercially available) that is included in each run.

### *Big* data types

**Fig. 1:**
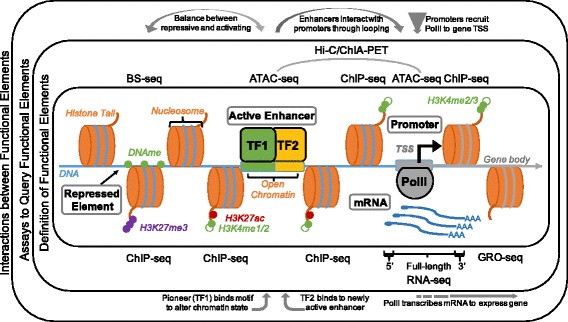
Common functional elements. Inner box: definitions of various functional elements (left to right): repressed element with methylated DNA (DNAme) and repressive modification of the histone tail of a nearby nucleosome (H3K27me3); active enhancer in an open chromatin region bound by two transcription factors (TF1, TF2) and marked by H3K4me1 or H3K4me2 (enhancer) and H3K27ac (activity); promoter bound by RNA Polymerase II (PolII) in an open chromatin region around the transcription start site (TSS, black arrow) of a gene body and marked by H3K4me2 or H3K4me3; and mRNA molecules transcribed from the gene with 5′ caps and 3′ poly (A) tails. Middle box: list of high-throughput sequencing assays used to annotate these functional elements, including bisulfite sequencing (BS-seq) for DNA methylation, Assay for Transposase Accessible Chromatin (ATAC-seq) for open chromatin, high-throughput chromosome conformation capture (Hi-C) or Chromatin Interaction Analysis by Paired-End Tag Sequencing (ChIA-PET) for interactions between regions, chromatin immunoprecipitation (ChIP-seq) for histone modifications, RNA-sequencing (RNA-seq) for mature mRNA (either 5′ biased, full length, or 3′ biased), and Global Run-On (GRO-seq) for nascent mRNA. Outer box: interactions between functional elements

High-throughput sequencing assays come in multiple flavors depending on the feature being measured. Regardless of the assay, the output is a library of DNA fragments or reads that are enriched for the feature of interest, whether that is expressed genes or epigenetically modified regions. A functional genomics study may combine two or more different assays on the same samples in order to gain a better picture of the underlying GRN. The relationship between different data types and their biological significance is shown in Fig. 1.

#### Gene expression

The expression level of a gene is determined by the number of times the DNA sequence of the gene is transcribed by RNA Polymerase II (PolII) into RNA and can be thought of as the output of GRNs. In the past, microarrays were commonly used to measure gene expression, but, more recently, RNA-seq has taken over as a more exact digital measurement for clinical applications [41, 42]. Typical RNA-seq measures the steady-state number of transcripts for each gene and may be full length with reads covering the whole sequence of the gene or end biased with reads starting at only the 5′ or 3′ end of the gene. Both methods have their advantages and disadvantages, with full-length reads allowing for analysis of allele-specific expression and alternative splicing, while end-biased reads are typically cheaper and provide a more accurate quantification of expression level [42]. Recently, advances in RNA-seq have allowed for high-throughput processing of samples with low cell numbers, such as one used on hematopoietic progenitors [36]. In addition, Global Run-on sequencing (GRO-seq) and similar assays were developed to measure nascent transcripts as they are transcribed and enrich for noncoding RNAs that play regulatory roles in the GRNs [43].

#### DNA methylation

DNA methylation, which primarily occurs on the cytosine (C) base of CpG sites, is an epigenetic mechanism associated with gene regulation. Genome-wide DNA methylation is typically measured by bisulfite sequencing (BS-seq), where bisulfite selectively converts unmethylated cytosine (C) into uracil that is then read by the sequencer as aberrant thymine (T) [44]. The biological significance arises from sites that are differentially methylated between cell types or conditions [45]. Extended regions of differential methylation (DMRs) between cell types are likely to occur in cis-regulatory elements and may reflect cell ontogeny [46]. In particular, regions enriched for CpGs, known as CpG islands, are often found around promoters and tend to be hypomethylated [45]. Methylation of CpG islands is associated with repressed genes; for example, a signature of methylation within these regions in fibroblast-like synoviocytes is thought to distinguish RA from osteoarthritis [47]. Moreover, differential methylation in blood is associated with genes in monozygotic twins who were discordant for SLE [48]. Outside of promoters, methylation has a more complex relationship with gene expression. For example, gene bodies may be heavily methylated in patterns that are associated with nucleosome positioning, binding of structural proteins (e.g., CTCF), intron–exon boundaries, and splicing [49, 50]. This dynamic is regulated by the opposing activity of DNA methyltransferases and demethylases whose expression may be altered in autoimmune disease, such as SLE [51]. Similarly, there is a close association between DNA methylation and histone modifications since hypermethylation tends to overlap repressive marks while hypomethylation is associated with activating marks at cis-regulatory elements [52].

#### Chromatin state

Along with DNA methylation, the genome-wide chromatin state of a cell is part of its epigenomic profile and may be used to annotate its cis-regulatory elements. When compared with gene expression, the chromatin state tends to give a broader picture of the cell’s GRN by highlighting the relevant interactors. Much of the genome consists of nucleosomes, approximately 150 bp of DNA wrapped around a histone octamer, that form the fundamental unit of chromatin [53]. Open chromatin, accessible regions of the genome that are depleted of nucleosomes, is associated with functional regulatory elements [54, 55]. The Assay for Transposase Accessible Chromatin (ATAC-seq) has emerged as an easy and versatile protocol for identifying open chromatin regions at low cell numbers [56]. Furthermore, specific post-translational modifications to histone tails have been associated with different regulatory elements [57]. The first genome-wide study of multiple histone modifications in human CD4^+^ T cells showed that, among other marks, variable levels of methylation of the fourth lysine on histone 3 were associated with different cis-regulatory elements: tri-methylation (H3K4me3) was highest at proximal elements, known as promoters, while mono-methylation (H3K4me1) was more commonly found near distal enhancers. H3K4me2 acts as a hybrid and has been used with other marks and gene contexts to mark both promoters or enhancers [12, 13]. Histone acetylation is typically associated with gene activation: for example, the combination of H3K27ac and H3K4me1 marks active enhancers while H3K4me1 alone is a mark of poised enhancers [58]. In contrast, H3K27me3 is found at repressed elements or, in combination with H3K4me3, bivalent domains, that occur specifically at the promoters of genes poised in stem cells [59, 60]. Together, the profiles of multiple histone modifications can be used to infer the chromatin state of genomic regions: in fact, ChromHMM was designed to automate this process using a machine-learning algorithm [61]. Chromatin immunoprecipitation (ChIP-seq) is used to generate a genome-wide map of these histone modifications, as well as the binding of specific TFs, based on the affinity of antibodies. Gosselin et al. [12] combined ChIP-seq of histone modifications and the myeloid TF, PU.1, to identify tissue-specific factors in macrophages. These chromatin assays have not yet been widely used in rheumatology: this is likely because of the inability to collect sufficient cell numbers in the models of interest. A recently developed protocol of ChIP-seq, named indexing-first ChIP (iChIP), enables parallelization of multiple modifications and samples with low input [36].

#### Chromatin interactions

In addition to the chromatin state of an individual region, it can be informative to assay the interactions of that region with the rest of the genome. Interacting regions include enhancer-to-promoter loops and topologically associated domains (TADs), which are thought to bring together distant genomic regions that are coregulated [62]. This three-dimensional structure of the chromatin has been widely assayed with Hi-C [63], a high-throughput successor of earlier chromosome conformation capture (3C) methods, and Chromatin Interaction Analysis by Paired-End Tagging (ChIA-PET) [64], an extension of ChIP to capture chromatin interactions. These technologies are at the forefront of innovation with new development in both experimental and computational approaches being put forward regularly. A new method, known as genome architecture mapping, represents an innovative approach to mapping chromatin interactions based on the principle that interacting regions should be frequently found in the same nuclear slice [65].

#### Single-cell technology

As opposed to the bulk assays already described that represent whole cell populations, single-cell technologies are aimed at assaying individual cells. The goal of single-cell approaches is to discern the heterogeneity of a population of cells without relying on *a priori* sorting. Thus, unlike the bulk assays, single-cell samples should be designed to contain a variable population. Typically, this variation is used to identify subpopulations of cells, which are then compared to reference bulk data sets or isolated as input for future assays. In particular, single-cell RNA-seq, which may be called scRNA-seq generically, has become quite popular in many fields including immunology [66], although other single-cell functional genomic protocols exist [67–71]. There are high hopes for the applicability of scRNA-seq to clinical rheumatology and precision medicine in the near future [66]. Single-cell protocols are generally just scaled-down versions of bulk protocols that are adapted for very low input. A few different versions of scRNA-seq exist that vary by the exact RNA-seq protocol (i.e., end biased or full length), the isolation of single cells, and the use of unique molecular identifiers (UMIs). For example, MARS-seq [40] and SMART-seq2 [37] both use fluorescence-activated cell sorting (FACS) to isolate single cells and deposit them in the wells of a cell-capture plate. While SMART-seq2 provides the additional information of full-length reads, MARS-seq uses 3′-biased reads with UMIs, which reduce noise by enabling the discrimination of true biological copies from amplification duplicates. Other versions of scRNA-seq rely on microfluidics (CEL-seq2 [72]) or microdroplets (Drop-seq [73]) to ensure single-cell reactions. Because of technical and biological limitations, single-cell technologies tend to have low coverage and depth, leading to sparse data sets and high noise [74]: thus, they will likely fall short of modeling the GRNs of individual cells. However, single-cell approaches are effective at assigning cells to different GRNs based on clustering or other algorithms that organize cells by similar cellular states, especially when used in combination with bulk assays or databases. These cellular states may differ based on cell type [37], development [40], cell cycle [75], spatial niche [76], response to stimuli [77], and so forth. For example, single-cell analysis of old and young hematopoietic stem cells demonstrated fewer old cells in the G1 phase and an inverse relationship between differentiation and age [75]. At present, single-cell analysis is fairly complicated owing to the large, complex data sets, but the bioinformatics support is rapidly catching up.

### *Big* bioinformatics

**Fig. 2:**
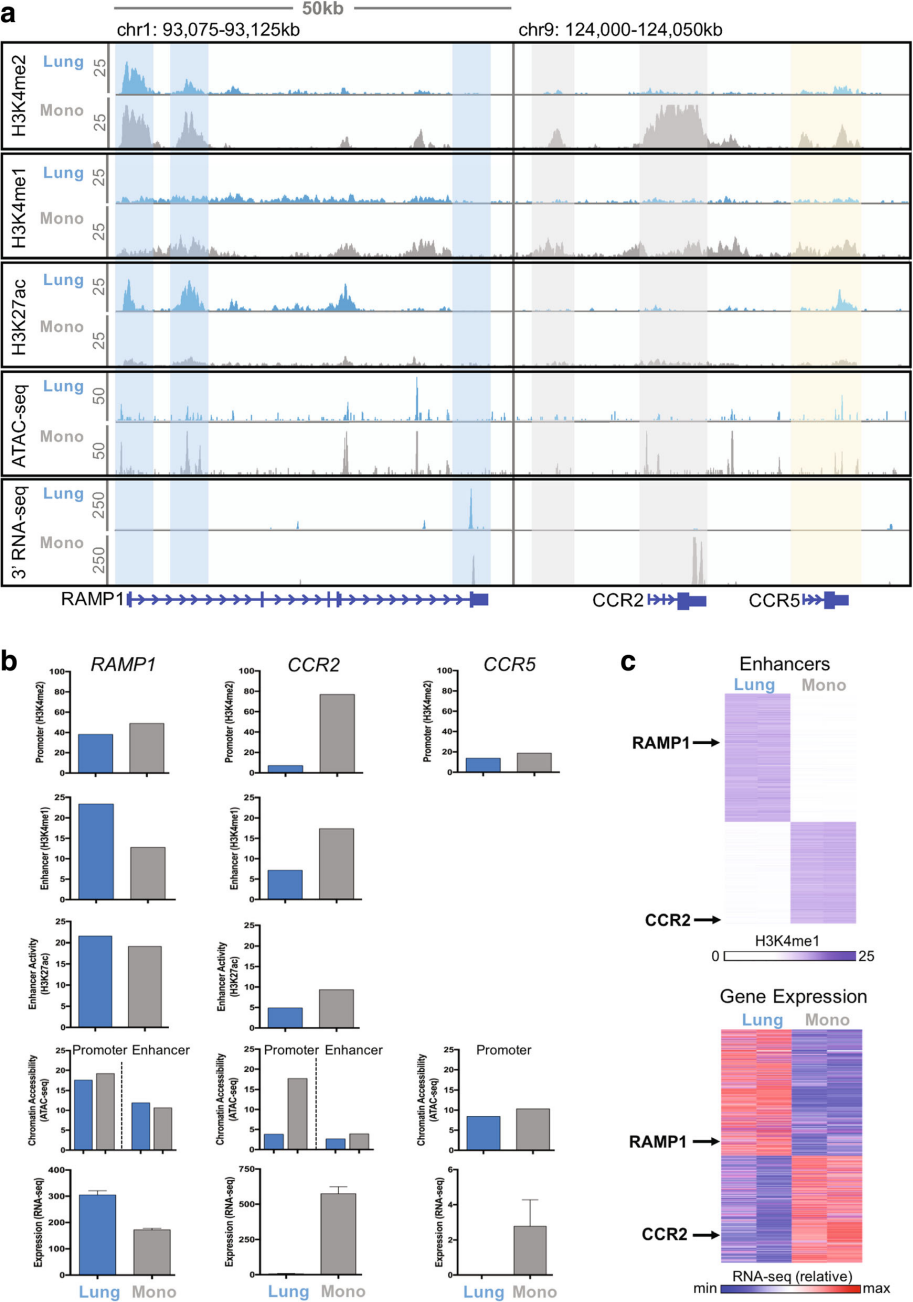
Sample output from functional genomic assays. Data generated from lung alveolar macrophages (blue) and bone marrow monocytes (grey) isolated from mice [13]. **a** Genome browser view of raw data from ChIP-seq (H3K4me2, H3K4me1, H3K27ac), ATAC-seq, and 3′-biased RNA-seq in 50 kb locus around RAMP1 and CCR2/CCR5. Highlighted regions from left to right represent: promoter, active intragenic enhancer, and 3′ end of RAMP1 (blue); poised intergenic enhancer and promoter/3′ end of CCR2 (gray); and promoter/3′ end of CCR2 (yellow). Genomic coordinates given above and scale of each track indicated on the left. Genes represented by blue lines below: thin lines for introns, medium lines for untranslated regions (UTRs), thick lines for exons; arrows on the gene body specify gene direction. **b** Quantitative measures of functional elements in **a**. Promoter usage is given by H3K4me2, enhancer usage by H3K4me1, enhancer activity by H3K27ac, chromatin accessibility by ATAC-seq, and gene expression by RNA-seq. Values represent normalized density (read count per kb region length per million reads) for ATAC-seq and ChIP-seq, and normalized CPM (counts per million reads) for RNA-seq (note varying scale). RAMP1, example of lung-specific gene with constitutive promoter. CCR2, example of highly monocyte-specific gene with monocyte-specific promoter and enhancer. CCR5, example of nonexpressed gene with low promoter activity. **c** Heatmaps clustered into lung-specific and monocyte-specific functional elements indicating how data from individual genes are integrated into global analyses. Differential enhancer usage measured by absolute value of H3K4me1 in 6575 regions and differential gene expression measured by relative value of RNA-seq in 3348 genes. ATAC-seq Assay for Transposase Accessible Chromatin followed by high-throughput sequencing, RNA-seq RNA-sequencing

The utility of high-throughput sequencing assays is dependent on the ability to analyze and model the resulting data. Fortunately, development of bioinformatic methods has progressed steadily, giving rise to a variety of standard techniques and publicly available software for the common approaches. For conciseness, this review begins with an analysis of the processed data (see Table 1) assuming that alignment, normalization, and filtering has occurred as necessary. In fact, these early processing steps are well covered by the literature and the related algorithms have already undergone several iterations of development since the dawn of high-throughput genomic assays. Readers are encouraged to approach the analysis as an extension of the experiment in which the various steps must be optimized, reproducible, and confirmed by multiple pieces of evidence. As in “wet” laboratory protocols, there is often no universally correct choice: the parameters must be decided based on the question and the data. The results should be presented given the implicit assumptions of the methods. Figure 2 shows sample output from RNA-seq, ATAC-seq, and ChIP-seq to give the reader an idea of what the data look like.

**Table 1:** Different assays for functional genomics, common protocols, format of the data after processing, and application to the GRN

Assay	Protocol	Processed data format	Direct application to GRN
Gene expression	RNA-seq, microarray	Table of samples (columns) by genes (rows)	Output
Chromatin state	ChIP-seq, ATAC-seq	List of peaks	Nodes
Chromatin interactions	Hi-C, ChIA-PET, GAM	Matrix of interactions	Edges
DNA methylation	BS-seq	List of methylated sites	Nodes
Single-cell technology	scRNA-seq	Table of cells (columns) by genes (rows)	Discriminate different GRNs

*GRN* gene regulatory network, *RNA*-*seq* RNA-sequencing, *ChIP*-*seq* chromatin immunoprecipitation followed by high-throughput sequencing, *ATAC*-*seq* Assay for Transposase Accessible Chromatin followed by high-throughput sequencing, *Hi*-*C* high-throughput chromosome conformation capture, *ChIA*-*PET* Chromatin Interaction Analysis by Paired-End Tag Sequencing, *GAM* genome architecture mapping, *BS*-*seq* bisulfite sequencing, *scRNA*-*seq* single-cell RNA-sequencing

#### Differential analysis

The simplest analysis involves a pairwise comparison between two samples or sets of samples. In the field of rheumatology, this will often, but not always, involve comparison of healthy to diseased cells. For gene expression, several algorithms exist to test, for each gene, the null hypothesis that the expression across the replicates of two samples is derived from the same distribution [78]. Because thousands of genes are compared in parallel, these algorithms often report a FDR to account for multiple hypotheses. In SSc, one might ask which genes are differentially expressed between fibrotic patients vs healthy control tissue [31] or treated vs untreated patients [33]. For DNA methylation, DMRs reflect changes in the methylation rate across windows covering many individual methylation sites [44]. For example, changes in the methylation status between T cells in lupus compared with health are thought to reflect changes to the regulation of adaptive immunity [51]. Pairwise comparison of peaks, regions of enrichment for the feature of interest, reflecting the chromatin state is less well defined. Complexity arises when determining which regions to compare since the peaks calls from one sample will not necessarily align with those in another. Simply assessing overlap of peaks is vulnerable to thresholding issues and differing levels of signal-to-noise ratios across samples: thus, peaks across samples are generally merged and the relevant scores are recalculated for the merged regions. Differential chromatin state reflects the opening, closing, or activation of cis-regulatory elements between samples, but these patterns may be more complicated than can be captured by a pairwise comparison.

#### Beyond pairwise comparisons

Often, differential analysis is not sufficient to cover the diversity or dimensionality of the samples in rheumatology. This may be the case when comparing multiple patient samples with disease subtypes [22] or when collecting multiple samples from different tissues or differentiation states [13, 36]. In these cases, supervised classification may be used when the labeling (e.g., clinical subtype) of samples is known. Otherwise, clustering provides an unsupervised option that does not rely on prior assumptions in order to address the question. Both samples and genes may be clustered based on their similarity by Pearson’s correlation, although there are alternative distance metrics. Milano et al. [22] used hierarchical clustering to group SSc patients by intrinsic gene expression. In contrast, *k*-means clustering was used to group enhancers based on H3K4me1 intensity across hematopoiesis [36]. Clustering of genes can also be used to categorize the coregulation of genes or regulatory elements. ImmGen executed a substantial effort of this sort to identify modules of coregulated genes across numerous immune cell types and link them to candidate regulators [79]. In human disease, we used the correlation of expression across macrophage samples isolated from the joints of RA patients to identify gene modules that may be associated with disease subtype (Mandelin et al, accepted). Similarly, Olsson et al. [80] use an iterative filtering approach to identify the most coregulated genes for clustering of single-cell data. Alternatively, assignment to meta-genes (or regulatory profiles) can be used to capture the major patterns of regulation across conditions, such as that seen by non-negative matrix factorization of gene expression through microglia development [40]. As the data sets get larger, dimension reduction methods enable visualization of samples as points in a graph of two-dimensional space. For instance, principal components analysis (PCA) of endogenous tissue-resident macrophage populations compared with those differentiated from bone marrow transplants after irradiation demonstrated the proximity of the chimera cells to their reference populations [13]. In addition, t-distributed stochastic neighbor embedding (tSNE) is commonly used for visualization of single-cell data, as in the dissection of DC and monocyte subpopulations in blood [37]. In other cases, it may be useful to compare the samples of interest to related data in order to better demarcate the genes involved. By including additional samples, researchers identified a consistent interferon signature between SSc and SLE blood samples [81] and distinguished between pulmonary fibrosis and hypertension in SSc through comparison with unrelated interstitial lung disease samples [23]. Alternatively, a meta-analysis may be used to indirectly compare the relevant genes between data sets; for example, to determine how well a mouse model of lupus reflects human disease [27] or to characterize which patients in a published cohort share a signature associated with imatinib response [82]. In practice, a combination of these techniques is used to address different questions and provide a robust analysis of the data. However, to fully capture the underlying GRNs, one must integrate different data types and use more sophisticated approaches.

#### Modeling gene regulatory networks

The purpose of modeling GRNs is to understand the overall scheme for the observed changes between samples. Typically, this means integrating data from a variety of sources on expression, methylation, and chromatin state. While individual changes in any one of these profiles may be subject to random change and not conserved by evolution, the global shape of the GRN should be fundamental to the identity of the cell. The nodes of the graph that constitute the GRN represent the cis-acting and trans-acting regulatory factors, while the edges represent interactions between them, such as the binding of TFs to regulatory elements or the looping of promoters and enhancers. Gene expression can be thought of as the output of these graphs; changes in expression indicate changes in the GRN, but may be of limited utility on their own in distinguishing between the many possible explanations. Given enough samples, gene expression may be used to implicate TF activity within a network as done by the Ontogenet algorithm developed by ImmGen to identify lineage-specific regulation [7] and by a network approach to a meta-analysis SSc cohort [28]. Alternatively, the chromatin state can provide additional depth and the ability to quantify the changes between conditions. By first choosing an assay to anchor the analysis—for example, peaks identified from the ATAC-seq data—one can then annotate these regions based on the remaining assays to identify promoters, poised enhancers, and active enhancers. The next step is to categorize the regions that change state between conditions. This method can be used to assess the degree of change across differentiation [83], environment [12, 13], or time [40]. One challenge in constructing the GRN is assigning cis-regulatory elements to the genes they regulate [84]. This is typically done by physical proximity, although this may not be reliable [55]. Another option is to use Hi-C or ChIA-PET data as proof of interactions between enhancers and gene promoters [63, 64]. Nodes at the apex of the GRN are enhancers or TFs that are involved in the regulation of other regulatory factors, such as lineage-determining TFs that specify the cell’s identity and pioneer TFs that bring about changes in the chromatin state [46]. Identification of these factors may be tantamount to defining the whole GRN. For example, identification of overrepresented TF binding motifs in the DNA sequence of enhancers based on their pattern of usage and expression of the associated TF family members has been used to implicate TFs associated with specification of cell types in hematopoiesis and environmental signals in tissue specificity [13, 36]. Similarly, by comparing gene expression and the dynamics of enhancers through T-cell development, Kitagawa et al. [83] implicated Satb1 in regulatory T-cell specification. The key factors of GRNs are likely to provide effective therapeutic targets depending on their relationship to disease.

#### Understanding the disease mechanism

Given a well-designed study, the differences in GRNs between samples should reflect the underlying disease mechanism. In effector cells, the GRN will explain how the cells are malfunctioning to enact disease and may even account for the cause, offering an attractive target for therapies. Sets of genes, whether arrived at through differential analysis, clustering, or other approach, may be compared to gene annotations to find enriched pathways or processes, as done to label gene modules in an SSc meta-analysis [28]. The gene ontology (GO) annotation is popularly used for this purpose, although it is limited by the underlying database (some genes may be unannotated) and biased by the more common cell types used to build this database. There has been some success in linking key sets of genes to disease severity [85] or response to treatment [33] using training (to identify the predictive genes) and testing (to validate their efficacy) cohorts. These approaches are likely to be improved with more data on cell-type-specific and tissue-specific populations. Moreover, changes in the GRN of a cell may be compared to publicly available databases to find similarities associated with shared triggers. For example, the LINCS website offers a similar function to recognize common signatures caused by specific cellular perturbations [6]. In rheumatic diseases with a genetic component, the causative single nucleotide polymorphisms (SNPs) are often found in noncoding regions where they have an impact on epigenomic regulation by directly or indirectly affecting TF binding [46]. When these SNPs are associated with changes in gene expression, termed eQTLs, they can be used to implicate cell types that are likely to play a role in disease, such as T-cell genes in RA and B cells in lupus [86, 87], although the underlying mechanism is not always clear. To uncover the molecular link between SNPs and disease, two studies which used data from genome-wide association studies (GWAS) and chromatin assays of multiple cell types found were able to implicate high-confidence causal SNPs that have the potential to disrupt regulatory elements [88–90]. But these studies, limited as they are by the sensitivity of GWAS and generic chromatin profiles from unrelated donors, are just the beginning.

### *Big* future

In the future, functional genomics approaches will become more prevalent in rheumatology. Certainly, the next review on a similar topic will have many more examples within the field on which to draw. Advances in other fields will influence how we view rheumatic disease and vice versa. As these assays become more popular, there are likely to be changes in their uses and scope.

#### Even bigger data

As the technology matures, the experiments will become cheaper and of even higher throughput. Rather than pairwise comparisons of test vs control, this will lead to larger data sets as more samples or single cells are assayed in a given experiment. In mouse models, this will enable more thorough modeling of GRNs by comparing the evolution of closely related populations at multiple conditions or time points. Human studies should include larger cohorts of patients and focus on identifying clinical subtypes in the pathogenesis of disease. Moreover, multiple forms of genomic data will be collected for each individual sample so that genetic variation can be directly associated with changes in the transcriptional and epigenomic profile. This is in contrast with the current situation where most epigenomic data from humans treat different individuals as replicates without the power to characterize individual variation. Similarly, whole tissue samples and in vitro cultures will be abandoned in favor of assays that focus on the cell type(s) of interest at multiple time points, states of differentiation/activation, or disease progression. With the advance in efficiency of assays, there will be a corresponding expansion in data available online.

#### Interactive public databases and user-friendly analysis programs

As already described, several concerted efforts already exist to collect data sets for public consumption. Currently, they are limited in the scope of the samples and the usefulness to the average researcher. Although most journals require authors to submit their data to a publicly available database, most biologists do not have the skills, time, or interest to find and use this raw data. In the future, when these databases reach a critical mass in the field of rheumatology, it will increasingly become more valuable for researchers to interact with them. Likewise, the databases will evolve to offer more user-friendly interfaces so that even the less tech-savvy users can find the information for which they are searching. Moreover, as the technology matures, increasingly more programs have become available that allow amateurs to peruse data and perform conventional analyses. Online programs to identify enriched GO terms and pathways are particularly prevalent, even for intergenic genomic regions [84]. There is even software with graphical user interfaces for sequencing pipelines (https://usegalaxy.org/) and clustering (https://software.broadinstitute.org/morpheus/). The challenge here is that they can be so easy to run that the user is unaware of the assumptions being made: this is why they do not replace training in functional genomics analysis. As the amount of data available increases and the bioinformatics methods become popularized, the skill set of researchers will evolve in kind.

#### Integration of computational approaches

At some point in the future, the distinction between computational biologists and other biologists will disappear. Already, young scientists are being taught quantitative methods and how to use bioinformatics tools as part of their general training in biology. In the future, a degree in biology will de facto include an education on computer science and statistics. As the average level of computational aptitude is raised, researchers will handle their own data throughout the processing and analysis stages. The bioinformatics capabilities of biologists and the accessibility of computational tools will dovetail to empower a progressively larger proportion of the community. Any competent researcher in functional genomics will be proficient at both the “wet” and “dry” components, meaning that advances in analysis will be synonymous with advances in experimental technique. As a result, the field of computational biology will become obsolete along with the value of so-called bioinformatics cores. Research in the intersection of rheumatology and genomics will be performed by interdisciplinary researchers or equal collaborations between rheumatologists and genomic investigators specializing in the cell type or tissue of interest.

## Conclusions

The goal of this review is to provide an overview of functional genomics and the challenges and possibilities inherent in big data. This overview comes at a time when genomics assays are widespread throughout biological fields but have yet to be fully taken advantage of in the field of rheumatology. This may be because it is not obvious to researchers how to make use of these data in a manner that extends beyond an exploratory study with descriptive results, or it may be because researchers are overwhelmed by the plethora of options and do not know where to begin. Therefore, the review has broken down functional genomics research into several stages. First, designing the study to best reflect the question of interest. Next, understanding the diverse data types that can be integrated into the following analysis. The bioinformatics section covers multiple approaches to be used in tandem to better model the underlying GRNs of disease. Finally, the review offers a future vision of the inevitable expansion of functional genomics into rheumatology.

## Abbreviations

ATAC-seq: Assay for Transposase Accessible Chromatin followed by high-throughput sequencing
BMDM: Bone-marrow-derived macrophage
BS-seq: BiSulfite sequencing
ChIA-PET: Chromatin Interaction Analysis by Paired-End Tag Sequencing
ChIP-seq: Chromatin Immuno Precipitation followed by high-throughput sequencing
DC: Dendritic cell
DMR: Differentially methylated regions
ENCODE: ENCyclopedia of DNA Elements
FDR: False discovery rate
GAM: Genome architecture mapping
GMCSF: Granulocyte–macrophage colony-stimulating factors
GRN: Gene regulatory network
GRO-seq: Global Run-On sequencing
GTex: Genotype-Tissue Expression project
GWAS: Genome-wide association study
Hi-C: High-throughput chromosome conformation capture
iChIP: Indexing first ChIP-seq
ImmGen: Immunological Genome Project
LINCS: Library of Integrated Network-based Cellular Signatures
PCA: Principal components analysis
PolII: RNA Polymerase II
RA: Rheumatoid arthritis
SNP: Single nucleotide polymorphism
SSc: Systemic sclerosis
STIA: Serum-transfer induced arthritis
TAD: Topologically associated domains
TF: Transcription factor

## References

[1] Consortium EP, Bernstein B, Birney E, Dunham I, Green E, Gunter C, Snyder M (2012). An integrated encyclopedia of DNA elements in the human genome. Nature.

[2] Consortium EP, Birney E, Stamatoyannopoulos J, Dutta A, Guigó R, Gingeras T, Margulies E, Weng Z, Snyder M, Dermitzakis E (2007). Identification and analysis of functional elements in 1% of the human genome by the ENCODE pilot project. Nature.

[3] Kundaje A, Meuleman W, Ernst J, Bilenky M, Yen A, Heravi-Moussavi A, Kheradpour P, Zhang Z, Wang J, Ziller MJ (2015). Integrative analysis of 111 reference human epigenomes. Nature.

[4] Consortium EP (2011). A user’s guide to the encyclopedia of DNA elements (ENCODE). PLoS Biol.

[5] Mele M, Ferreira PG, Reverter F, DeLuca DS, Monlong J, Sammeth M, Young TR, Goldmann JM, Pervouchine DD, Sullivan TJ (2015). Human genomics. The human transcriptome across tissues and individuals. Science.

[6] Duan Q, Flynn C, Niepel M, Hafner M, Muhlich JL, Fernandez NF, Rouillard AD, Tan CM, Chen EY, Golub TR (2014). LINCS Canvas Browser: interactive web app to query, browse and interrogate LINCS L1000 gene expression signatures. Nucleic Acids Res.

[7] Heng TS, Painter MW, Immunological Genome Project Consortium (2008). The Immunological Genome Project: networks of gene expression in immune cells. Nat Immunol.

[8] Martens JHA, Stunnenberg HG (2013). BLUEPRINT: mapping human blood cell epigenomes. Haematologica.

[9] Ostuni R, Piccolo V, Barozzi I, Polletti S, Termanini A, Bonifacio S, Curina A, Prosperini E, Ghisletti S, Natoli G (2013). Latent enhancers activated by stimulation in differentiated cells. Cell.

[10] Heinz S, Benner C, Spann N, Bertolino E, Lin Y, Laslo P, Cheng J, Murre C, Singh H, Glass C (2010). Simple combinations of lineage-determining transcription factors prime cis-regulatory elements required for macrophage and B cell identities. Mol Cell.

[11] Helft J, Bottcher J, Chakravarty P, Zelenay S, Huotari J, Schraml BU, Goubau D (2015). Reis e Sousa C. GM-CSF mouse bone marrow cultures comprise a heterogeneous population of CD11c(+)MHCII(+) macrophages and dendritic cells. Immunity.

[12] Gosselin D, Link VM, Romanoski CE, Fonseca GJ, Eichenfield DZ, Spann NJ, Stender JD, Chun HB, Garner H, Geissmann F (2014). Environment drives selection and function of enhancers controlling tissue-specific macrophage identities. Cell.

[13] Lavin Y, Winter D, Blecher-Gonen R, David E, Keren-Shaul H, Merad M, Jung S, Amit I (2014). Tissue-resident macrophage enhancer landscapes are shaped by the local microenvironment. Cell.

[14] Gardner H, Shearstone JR, Bandaru R, Crowell T, Lynes M, Trojanowska M, Pannu J, Smith E, Jablonska S, Blaszczyk M (2006). Gene profiling of scleroderma skin reveals robust signatures of disease that are imperfectly reflected in the transcript profiles of explanted fibroblasts. Arthritis Rheum.

[15] Bessis N, Decker P, Assier E, Semerano L, Boissier MC (2017). Arthritis models: usefulness and interpretation. Semin Immunopathol.

[16] Marangoni RG, Varga J, Tourtellotte WG (2016). Animal models of scleroderma: recent progress. Curr Opin Rheumatol.

[17] Bender AT, Wu Y, Cao Q, Ding Y, Oestreicher J, Genest M, Akare S, Ishizaka ST, Mackey MF (2014). Assessment of the translational value of mouse lupus models using clinically relevant biomarkers. Transl Res.

[18] Rose S, Eren M, Murphy S, Zhang H, Thaxton CS, Chowaniec J, Waters EA, Meade TJ, Vaughan DE, Perlman H (2013). A novel mouse model that develops spontaneous arthritis and is predisposed towards atherosclerosis. Ann Rheum Dis.

[19] Bethunaickan R, Berthier CC, Ramanujam M, Sahu R, Zhang W, Sun Y, Bottinger EP, Ivashkiv L, Kretzler M, Davidson A (2011). A unique hybrid renal mononuclear phagocyte activation phenotype in murine systemic lupus erythematosus nephritis. J Immunol.

[20] Mouratis MA, Aidinis V (2011). Modeling pulmonary fibrosis with bleomycin. Curr Opin Pulm Med.

[21] Misharin AV, Morales-Nebreda L, Reyfman PA, Cuda CM, Walter JM, McQuattie-Pimentel AC, Chen CI, Anekalla KR, Joshi N, Williams KJN (2017). Monocyte-derived alveolar macrophages drive lung fibrosis and persist in the lung over the life span. J Exp Med.

[22] Milano A, Pendergrass SA, Sargent JL, George LK, McCalmont TH, Connolly MK, Whitfield ML (2008). Molecular subsets in the gene expression signatures of scleroderma skin. PLoS One.

[23] Hsu E, Shi H, Jordan RM, Lyons-Weiler J, Pilewski JM, Feghali-Bostwick CA (2011). Lung tissues in patients with systemic sclerosis have gene expression patterns unique to pulmonary fibrosis and pulmonary hypertension. Arthritis Rheum.

[24] Bharat A, Bhorade SM, Morales-Nebreda L, McQuattie-Pimentel AC, Soberanes S, Ridge K, DeCamp MM, Mestan KK, Perlman H, Budinger GR (2016). Flow cytometry reveals similarities between lung macrophages in humans and mice. Am J Respir Cell Mol Biol.

[25] Misharin AV, Cuda CM, Saber R, Turner JD, Gierut AK, Haines GK, Berdnikovs S, Filer A, Clark AR, Buckley CD (2014). Nonclassical Ly6C(-) monocytes drive the development of inflammatory arthritis in mice. Cell Rep.

[26] Bernstein BE, Kamal M, Lindblad-Toh K, Bekiranov S, Bailey DK, Huebert DJ, McMahon S, Karlsson EK, Kulbokas EJ, Gingeras TR (2005). Genomic maps and comparative analysis of histone modifications in human and mouse. Cell.

[27] Berthier CC, Bethunaickan R, Gonzalez-Rivera T, Nair V, Ramanujam M, Zhang W, Bottinger EP, Segerer S, Lindenmeyer M, Cohen CD (2012). Cross-species transcriptional network analysis defines shared inflammatory responses in murine and human lupus nephritis. J Immunol.

[28] Mahoney JM, Taroni J, Martyanov V, Wood TA, Greene CS, Pioli PA, Hinchcliff ME, Whitfield ML (2015). Systems level analysis of systemic sclerosis shows a network of immune and profibrotic pathways connected with genetic polymorphisms. PLoS Comput Biol.

[29] Altboum Z, Steuerman Y, David E, Barnett-Itzhaki Z, Valadarsky L, Keren-Shaul H, Meningher T, Mendelson E, Mandelboim M, Gat-Viks I (2014). Digital cell quantification identifies global immune cell dynamics during influenza infection. Mol Syst Biol.

[30] Becht E, Giraldo NA, Lacroix L, Buttard B, Elarouci N, Petitprez F, Selves J, Laurent-Puig P, Sautes-Fridman C, Fridman WH (2016). Estimating the population abundance of tissue-infiltrating immune and stromal cell populations using gene expression. Genome Biol.

[31] Christmann RB, Sampaio-Barros P, Stifano G, Borges CL, de Carvalho CR, Kairalla R, Parra ER, Spira A, Simms R, Capellozzi VL (2014). Association of Interferon- and transforming growth factor beta-regulated genes and macrophage activation with systemic sclerosis-related progressive lung fibrosis. Arthritis Rheumatol.

[32] Higashi-Kuwata N, Jinnin M, Makino T, Fukushima S, Inoue Y, Muchemwa FC, Yonemura Y, Komohara Y, Takeya M, Mitsuya H (2010). Characterization of monocyte/macrophage subsets in the skin and peripheral blood derived from patients with systemic sclerosis. Arthritis Res Ther.

[33] Hinchcliff M, Huang CC, Wood TA, Matthew Mahoney J, Martyanov V, Bhattacharyya S, Tamaki Z, Lee J, Carns M, Podlusky S (2013). Molecular signatures in skin associated with clinical improvement during mycophenolate treatment in systemic sclerosis. J Invest Dermatol.

[34] Ching T, Huang S, Garmire LX (2014). Power analysis and sample size estimation for RNA-Seq differential expression. RNA.

[35] Lee ML, Whitmore GA (2002). Power and sample size for DNA microarray studies. Stat Med.

[36] Lara-Astiaso D, Weiner A, Lorenzo-Vivas E, Zaretsky I, Jaitin D, David E, Keren-Shaul H, Mildner A, Winter D, Jung S (2014). Chromatin state dynamics during blood formation. Science.

[37] Villani AC, Satija R, Reynolds G, Sarkizova S, Shekhar K, Fletcher J, Griesbeck M, Butler A, Zheng S, Lazo S et al. Single-cell RNA-seq reveals new types of human blood dendritic cells, monocytes, and progenitors. Science. 2017;356(6335):eaah4573. 10.1126/science.aah4573 PMC5775029 28428369

[38] Leek J, Scharpf R, Bravo H, Simcha D, Langmead B, Johnson W, Geman D, Baggerly K, Irizarry R (2010). Tackling the widespread and critical impact of batch effects in high-throughput data. Nat Rev Genet.

[39] Oytam Y, Sobhanmanesh F, Duesing K, Bowden JC, Osmond-McLeod M, Ross J (2016). Risk-conscious correction of batch effects: maximising information extraction from high-throughput genomic datasets. BMC Bioinf.

[40] Matcovitch-Natan O, Winter DR, Giladi A, Vargas Aguilar S, Spinrad A, Sarrazin S, Ben-Yehuda H, David E, Zelada González F, Perrin P (2016). Microglia development follows a stepwise program to regulate brain homeostasis. Science.

[41] Zhang W, Yu Y, Hertwig F, Thierry-Mieg J, Zhang W, Thierry-Mieg D, Wang J, Furlanello C, Devanarayan V, Cheng J (2015). Comparison of RNA-seq and microarray-based models for clinical endpoint prediction. Genome Biol.

[42] Conesa A, Madrigal P, Tarazona S, Gomez-Cabrero D, Cervera A, McPherson A, Szczesniak MW, Gaffney DJ, Elo LL, Zhang X (2016). A survey of best practices for RNA-seq data analysis. Genome Biol.

[43] Lopes R, Agami R, Korkmaz G (2017). GRO-seq, a tool for identification of transcripts regulating gene expression. Methods Mol Biol.

[44] Beck S, Rakyan V (2008). The methylome: approaches for global DNA methylation profiling. Trends Genet.

[45] Viatte S, Plant D, Raychaudhuri S (2013). Genetics and epigenetics of rheumatoid arthritis. Nat Rev Rheumatol.

[46] Winter DR, Jung S, Amit I (2015). Making the case for chromatin profiling: a new tool to investigate the immune-regulatory landscape. Nat Rev Immunol.

[47] Nakano K, Whitaker JW, Boyle DL, Wang W, Firestein GS (2013). DNA methylome signature in rheumatoid arthritis. Ann Rheum Dis.

[48] Javierre BM, Fernandez AF, Richter J, Al-Shahrour F, Martin-Subero JI, Rodriguez-Ubreva J, Berdasco M, Fraga MF, O’Hanlon TP, Rider LG (2010). Changes in the pattern of DNA methylation associate with twin discordance in systemic lupus erythematosus. Genome Res.

[49] Chodavarapu R, Feng S, Bernatavichute Y, Chen P-Y, Stroud H, Yu Y, Hetzel J, Kuo F, Kim J, Cokus S (2010). Relationship between nucleosome positioning and DNA methylation. Nature.

[50] Shukla S, Kavak E, Gregory M, Imashimizu M, Shutinoski B, Kashlev M, Oberdoerffer P, Sandberg R, Oberdoerffer S (2011). CTCF-promoted RNA polymerase II pausing links DNA methylation to splicing. Nature.

[51] Hedrich CM, Mabert K, Rauen T, Tsokos GC (2017). DNA methylation in systemic lupus erythematosus. Epigenomics.

[52] Cedar H, Bergman Y (2009). Linking DNA methylation and histone modification: patterns and paradigms. Nat Rev Genet.

[53] Richmond T, Davey C (2003). The structure of DNA in the nucleosome core. Nature.

[54] Gross D, Garrard W (1988). Nuclease hypersensitive sites in chromatin. Annu Rev Biochem.

[55] Thurman R, Rynes E, Humbert R, Vierstra J, Maurano M, Haugen E, Sheffield N, Stergachis A, Wang H, Vernot B (2012). The accessible chromatin landscape of the human genome. Nature.

[56] Buenrostro J, Giresi P, Zaba L, Chang H, Greenleaf W (2013). Transposition of native chromatin for fast and sensitive epigenomic profiling of open chromatin, DNA-binding proteins and nucleosome position. Nat Methods.

[57] Kouzarides T (2007). Chromatin modifications and their function. Cell.

[58] Creyghton M, Cheng A, Welstead G, Kooistra T, Carey B, Steine E, Hanna J, Lodato M, Frampton G, Sharp P (2010). Histone H3K27ac separates active from poised enhancers and predicts developmental state. Proc Natl Acad Sci U S A.

[59] Barski A, Cuddapah S, Cui K, Roh T-Y, Schones D, Wang Z, Wei G, Chepelev I, Zhao K (2007). High-resolution profiling of histone methylations in the human genome. Cell.

[60] Cui K, Zang C, Roh T-Y, Schones DE, Childs RW, Peng W, Zhao K (2009). Chromatin signatures in multipotent human hematopoietic stem cells indicate the fate of bivalent genes during differentiation. Cell Stem Cell.

[61] Ernst J, Kellis M (2012). ChromHMM: automating chromatin-state discovery and characterization. Nat Methods.

[62] Dixon J, Selvaraj S, Yue F, Kim A, Li Y, Shen Y, Hu M, Liu J, Ren B (2012). Topological domains in mammalian genomes identified by analysis of chromatin interactions. Nature.

[63] Lieberman-Aiden E, van Berkum N, Williams L, Imakaev M, Ragoczy T, Telling A, Amit I, Lajoie B, Sabo P, Dorschner M (2009). Comprehensive mapping of long-range interactions reveals folding principles of the human genome. Science.

[64] Kieffer-Kwon K-R, Tang Z, Mathe E, Qian J, Sung M-H, Li G, Resch W, Baek S, Pruett N, Grøntved L (2013). Interactome maps of mouse gene regulatory domains reveal basic principles of transcriptional regulation. Cell.

[65] Beagrie RA, Scialdone A, Schueler M, Kraemer DC, Chotalia M, Xie SQ, Barbieri M, de Santiago I, Lavitas LM, Branco MR (2017). Complex multi-enhancer contacts captured by genome architecture mapping. Nature.

[66] Jaitin DA, Keren-Shaul H, Elefant N, Amit I (2015). Each cell counts: hematopoiesis and immunity research in the era of single cell genomics. Semin Immunol.

[67] Buenrostro JD, Wu B, Litzenburger UM, Ruff D, Gonzales ML, Snyder MP, Chang HY, Greenleaf WJ (2015). Single-cell chromatin accessibility reveals principles of regulatory variation. Nature.

[68] Rotem A, Ram O, Shoresh N, Sperling RA, Goren A, Weitz DA, Bernstein BE (2015). Single-cell ChIP-seq reveals cell subpopulations defined by chromatin state. Nat Biotechnol.

[69] Farlik M, Sheffield NC, Nuzzo A, Datlinger P, Schonegger A, Klughammer J, Bock C (2015). Single-cell DNA methylome sequencing and bioinformatic inference of epigenomic cell-state dynamics. Cell Rep.

[70] Smallwood SA, Lee HJ, Angermueller C, Krueger F, Saadeh H, Peat J, Andrews SR, Stegle O, Reik W, Kelsey G (2014). Single-cell genome-wide bisulfite sequencing for assessing epigenetic heterogeneity. Nat Methods.

[71] Cusanovich DA, Daza R, Adey A, Pliner HA, Christiansen L, Gunderson KL, Steemers FJ, Trapnell C, Shendure J (2015). Multiplex single cell profiling of chromatin accessibility by combinatorial cellular indexing. Science.

[72] Hashimshony T, Senderovich N, Avital G, Klochendler A, de Leeuw Y, Anavy L, Gennert D, Li S, Livak KJ, Rozenblatt-Rosen O (2016). CEL-Seq2: sensitive highly-multiplexed single-cell RNA-Seq. Genome Biol.

[73] Macosko EZ, Basu A, Satija R, Nemesh J, Shekhar K, Goldman M, Tirosh I, Bialas AR, Kamitaki N, Martersteck EM (2015). Highly parallel genome-wide expression profiling of individual cells using nanoliter droplets. Cell.

[74] Kim JK, Kolodziejczyk AA, Ilicic T, Teichmann SA, Marioni JC (2015). Characterizing noise structure in single-cell RNA-seq distinguishes genuine from technical stochastic allelic expression. Nat Commun.

[75] Kowalczyk MS, Tirosh I, Heckl D, Rao TN, Dixit A, Haas BJ, Schneider RK, Wagers AJ, Ebert BL, Regev A (2015). Single-cell RNA-seq reveals changes in cell cycle and differentiation programs upon aging of hematopoietic stem cells. Genome Res.

[76] Halpern KB, Shenhav R, Matcovitch-Natan O, Toth B, Lemze D, Golan M, Massasa EE, Baydatch S, Landen S, Moor AE (2017). Single-cell spatial reconstruction reveals global division of labour in the mammalian liver. Nature.

[77] Gury-BenAri M, Thaiss CA, Serafini N, Winter DR, Giladi A, Lara-Astiaso D, Levy M, Salame TM, Weiner A, David E (2016). The spectrum and regulatory landscape of intestinal innate lymphoid cells are shaped by the microbiome. Cell.

[78] Zhang ZH, Jhaveri DJ, Marshall VM, Bauer DC, Edson J, Narayanan RK, Robinson GJ, Lundberg AE, Bartlett PF, Wray NR (2014). A comparative study of techniques for differential expression analysis on RNA-Seq data. PLoS One.

[79] Jojic V, Shay T, Sylvia K, Zuk O, Sun X, Kang J, Regev A, Koller D, Best AJ, Immunological Genome Project C (2013). Identification of transcriptional regulators in the mouse immune system. Nat Immunol.

[80] Olsson A, Venkatasubramanian M, Chaudhri VK, Aronow BJ, Salomonis N, Singh H, Grimes HL (2016). Single-cell analysis of mixed-lineage states leading to a binary cell fate choice. Nature.

[81] Assassi S, Mayes MD, Arnett FC, Gourh P, Agarwal SK, McNearney TA, Chaussabel D, Oommen N, Fischbach M, Shah KR (2010). Systemic sclerosis and lupus: points in an interferon-mediated continuum. Arthritis Rheum.

[82] Chung L, Fiorentino DF, Benbarak MJ, Adler AS, Mariano MM, Paniagua RT, Milano A, Connolly MK, Ratiner BD, Wiskocil RL (2009). Molecular framework for response to imatinib mesylate in systemic sclerosis. Arthritis Rheum.

[83] Kitagawa Y, Ohkura N, Kidani Y, Vandenbon A, Hirota K, Kawakami R, Yasuda K, Motooka D, Nakamura S, Kondo M (2017). Guidance of regulatory T cell development by Satb1-dependent super-enhancer establishment. Nat Immunol.

[84] McLean C, Bristor D, Hiller M, Clarke S, Schaar B, Lowe C, Wenger A, Bejerano G (2010). GREAT improves functional interpretation of cis-regulatory regions. Nat Biotechnol.

[85] Lofgren S, Hinchcliff M, Carns M, Wood T, Aren K, Arroyo E, Cheung P, Kuo A, Valenzuela A, Haemel A (2016). Integrated, multicohort analysis of systemic sclerosis identifies robust transcriptional signature of disease severity. JCI Insight.

[86] Hu X, Kim H, Raj T, Brennan PJ, Trynka G, Teslovich N, Slowikowski K, Chen WM, Onengut S, Baecher-Allan C (2014). Regulation of gene expression in autoimmune disease loci and the genetic basis of proliferation in CD4+ effector memory T cells. PLoS Genet.

[87] Hu X, Kim H, Stahl E, Plenge R, Daly M, Raychaudhuri S (2011). Integrating autoimmune risk loci with gene-expression data identifies specific pathogenic immune cell subsets. Am J Hum Genet.

[88] Farh KK-H, Marson A, Zhu J, Kleinewietfeld M, Housley WJ, Beik S, Shoresh N, Whitton H, Ryan RJH, Shishkin AA et al. Genetic and epigenetic fine mapping of causal autoimmune disease variants. Nature 2014, advance online publication. 10.1038/nature13835 PMC4336207 25363779

[89] Trynka G, Sandor C, Han B, Xu H, Stranger B, Liu X, Raychaudhuri S (2013). Chromatin marks identify critical cell types for fine mapping complex trait variants. Nat Genet.

[90] Hui-Yuen JS, Zhu L, Wong LP, Jiang K, Chen Y, Liu T, Jarvis JN (2016). Chromatin landscapes and genetic risk in systemic lupus. Arthritis Res Ther.

